# Relationship between transcutaneous C02 measurement and PAC02 during non invasive ventilation delivered because of hypercapnic acute respiratory failure

**DOI:** 10.1186/2197-425X-3-S1-A386

**Published:** 2015-10-01

**Authors:** D Thévoz, J-P Revelly, P Jolliet, L Piquilloud

**Affiliations:** Cardio-respiratory Physiotherapy Unit, University Hospital of Lausanne, Lausanne, Switzerland; Adult Intensive Care and Burn Unit, University Hospital of Lausanne, Lausanne, Switzerland

## Introduction

Non-invasive ventilation (NIV) is the first line supportive treatment in case of acute hypercapnic respiratory failure (AHRF). NIV efficacy is continuously monitored using clinical parameters (respiratory frequency, use of accessory respiratory muscles). The assessment of NIV efficacy however usually requires repeated blood gas analysis after 30 or 60 minutes of treatment. A reliable non-invasive technique to continuously monitor PaC02 during NIV could simplify this evaluation and allow an earlier adaptation of ventilator settings.

## Objectives

The aim of this study was to assess whether measuring transcutaneous C02 (T_p_C02) during NIV delivered because of AHRF could be of interest for evaluating PaC02.

## Methods

ICU patients requiring NIV for AHRF (PaC02 >42 mmHg) were included in this prospective observational study. T_p_C02 was measured during a 60-minute NIV treatment using a dedicated auricular sensor and the Sentec monitor (Sentec, Switzerland), connected to the patient 15 minutes before the start of NIV. Blood gas analysis and T_c_C02 recording were performed before initiating NIV and after 30, 45 and 60 minutes of NIV.The correlation between PaC02 and T_p_C02 was assessed by linear regression (Spearman) and intraclass correlation coefficient (ICC). The agreement between both techniques was assessed using the Bland and Altmann method for repeated measurements.

## Results

**Figure 1:**
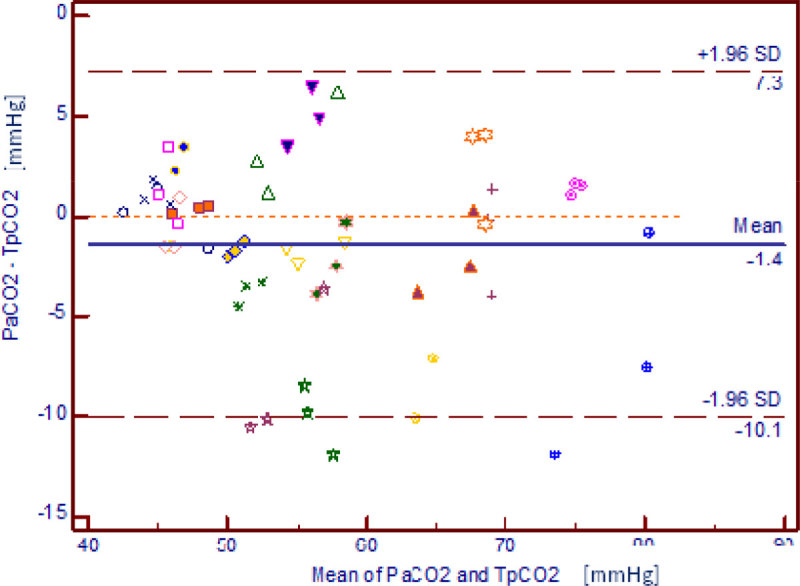
Figure 1

20 patients (11 women; 9 men, 17 with obstructive pulmonary disease, 1 with restrictive disease and 2 without chronic lung disease) were included in the study. Age 65 [61-72] years II score 32 [28-46]. At inclusion, PaC02 was 57 [51-68] mmHg, Sa02 94 [92-95] % and respiratory rate 25 [21-29] /min. PaC02 values ranged from 43 to 80 mmHg whereas T_p_C02 values ranged from 42 to 84 mmHg. The correlation coefficient RSAPS ^2^ between PaC02 and T_p_C02 values was 0.84. The ICC was 0.906. Bland and Altmann graph is illustrated in figure 1. The bias was -1.4 mmHg and the limits of agreement were -10.1 and 7.3 mmHg.

## Conclusions

In a small group of patients undergoing NIV for acute hypercapnic respiratory failure the agreement between T_p_C02 and PaC02 was very good. This suggests that C02 transcutaneous measurement could be of interest to evaluate the course of PaC02 during NIV.

